# A pilot study on the pulmonary anthracosis in stray dogs of Kathmandu Valley, Nepal: A potential public health threat for future

**DOI:** 10.14202/vetworld.2024.658-665

**Published:** 2024-03-21

**Authors:** Sunil Thapa, Rajesh Bhatta, Bikash Puri, Rajendra Bashyal, Romi Kunwar, Swochhal Prakash Shrestha, Girija Regmi, Pushkar Pal

**Affiliations:** 1 Department of Veterinary Pathology and Clinics, Agriculture and Forest University, Chitwan, 44202, Nepal; 2 KAT Centre, Kathmandu, 44600, Nepal; 3 Veterinary Public Health and Food Safety Centre for Asia Pacific, Faculty of Veterinary Medicine, Chiang Mai University, Chiang Mai, 50100, Thailand; 4 Tifton Veterinary Diagnostic and Investigational Laboratory, University of Georgia, Tifton, 31793 Georgia, USA

**Keywords:** air pollution, histopathology, Kathmandu, pulmonary anthracosis, respiratory disease, stray dogs

## Abstract

**Background and Aim:**

Kathmandu is a densely populated metropolitan city in Nepal. In recent years, however, the metropolis has been ranked as one of the most polluted cities worldwide. Both humans and animals are susceptible to various respiratory diseases due to chronic exposure to polluted air. Due to the relative similarities in the anatomical structure and physiological functions of the respiratory system between humans and dogs, polluted environments may lead to respiratory illness in similar ways in both species living in the valley. On the basis of information on the air quality in the valley, this study was conceived to assess pulmonary illness in street dogs to discern the health hazards caused by polluted air.

**Materials and Methods:**

A total of 76 dogs with clinical signs of tachypnea, dyspnea, sneezing, coughing, mucopurulent discharge, moderate hyperthermia, and anorexia admitted from July 2020 to November 2020 in Animal Nepal for treatment were included in this study. Among them, 24 animals responded to treatment, and 52 dogs died during their stay in the hospital. The 52 dead animals were necropsied, and the lesions that resembled pulmonary anthracosis were further studied grossly and histologically in a blinded fashion by trained veterinary pathologists.

**Results:**

Significant morphological alterations were observed in the lungs and associated lymph nodes of 25 animals, indicating pulmonary anthracosis. Gross morphological changes included multiple black foci with hemorrhage, congestion, nodular, and emphysema on the parietal and visceral surfaces of the lungs. The alveolar septa and visceral pleura exhibited deposition of black particles. Congestion, emphysema, and inflammatory exudates were also detected in the lung tissues and lymph nodes.

**Conclusion:**

The clinical, gross, and microscopic findings accurately resembled those of pulmonary anthracosis. This life-threatening condition in stray canines may be caused by a critical level of air pollution from different sources and carbon emissions from vehicles. To protect animals and humans living in the Kathmandu Valley, concerned government and non-government agencies should work toward reducing air pollution levels as soon as possible.

## Introduction

Air pollutants are any physical, chemical or biological substances that affect the atmospheric properties of the ambient air. It is a complicated mixture of thousands of different elements, which include different particulate matter (PM) and pollutants such as ozone, nitrogen dioxide, volatile organic compounds such as benzene, carbon monoxide (CO), and sulfur dioxide [1]. In addition to climate change, air pollution is one of the most serious environmental threats to human health. According to a report from the World Health Organization, every year, an estimated 7 million people are killed by air pollution due to respiratory and other diseases, with individuals in low- and middle-income countries most affected by outdoor air pollution [2]. It is ironic that anthropogenic activities contribute the greatest to this alarming global air pollution and subsequent high mortality [1].

Emissions from local sources, such as motor vehicles, brick kilns, biomass/garbage burning, and dust storms, are the main air pollutants in the Kathmandu Valley [1]. Kathmandu is witnessing rapid growth, with different infrastructure development projects implemented in a short period of time. The population of Kathmandu has been growing rapidly in the past 20 years due to the rapid urbanization process [3]. There has been a huge influx of people from remote areas looking for employment and other facilities that the city offers [4]. Although urbanization has some beneficial impacts on society, Kathmandu as a city is sustaining this high population with significant compromise in air quality and declining animal and human health induced by the poor ambient air quality. As a result, several problems, including environmental and health issues, are more common. Among these severe problems, contaminated environment, heavy traffic, and less vegetation have led to pulmonary disease in humans [5]. According to IQAir (Goldach, Switzerland), the Swiss group that manages real-time worldwide air quality data, Kathmandu’s air quality index (AQI) level was between “very unhealthy” and “hazardous” levels at the end of March 2021, with the highest AQI level recorded at 411 μg/cubic meter at 9:45 am on Saturday with PM 2.5 at 366 μg/cubic meter [6]. PM 2.5 refers to minute airborne particles or droplets with a width of 2 and a half microns or less [7]. PM 2.5 particles are considered hazardous because they can travel deep into the respiratory tract, reaching the lungs [8]. The intake of minute particles irritates the eyes, nose, throat, and lungs and causes coughing, sneezing, runny nose, and shortness of breath [9]. Long-term exposure to these minute air particles leads to various respiratory and cardiovascular morbidities, such as asthma, chronic obstructive pulmonary disease (COPD), cancer, and cardiovascular diseases. The Kathmandu Valley contains a ridiculous number of cars, which are one of the main sources of CO. When CO comes in contact with the blood, it causes carboxyhemoglobin, which causes headache, dizziness, disorientation, seizure, hypotension, arrhythmia, and pulmonary dysfunction [1].

The polluted environment in Kathmandu contains toxic substances that cause human and animal pulmonary diseases such as anthracosis [10]. Anthracosis is defined as the accumulation of carbon particles in the lungs [11] It is a milder form of pneumoconiosis caused by carbon deposition in the lungs caused by repeated exposure to air pollution or inhalation of smoke or coal dust particles [12]. Humans are more likely to contract anthracosis than animals because of dust exposure in jobs such as coal mining and manufacturing that produce fumes [13]. However, anthracosis is equally common in nearby animals; dogs, horses, and mules are particularly vulnerable [14]. Anthracosis has also been reported in cattle, birds, reptiles, and mammals kept in zoological parks [15].

Recent research has shown that the increased incidence and prevalence of non-communicable diseases such as diabetes, cancer, COPD, hypertension, and mental illness in the Kathmandu Valley may be directly correlated with poor ambient air quality [1]. Since stray dogs breathe the same air as humans in the Kathmandu Valley, it is also possible that these dogs may suffer from the same diseases as humans in the valley. In this context, the research aims to uncover similarities between animals, specifically stray dogs, and air pollution concerning human health. In addition to the huge human population in Kathmandu, stray dogs living in the open streets of Kathmandu are exposed to toxic environment [16]. Because air quality in Kathmandu is the most dangerous for living creatures, the stray dog population is also considered to be at high risk of anthracosis.

Thus, this study aimed to evaluate the impact of air pollution on street dogs by examining the canine population reported for respiratory illness in various animal welfare clinics. Furthermore, this study documents gross and microscopic lesions of respiratory illness in stray dogs, which can be studied side-by-side to determine the extent of pulmonary illness caused by air pollution in humans.

## Materials and Methods

## Ethical approval

Ethical approval for the study was not required because the samples were collected from dead animals from the canine shelter of Animal Nepal.

### Study period and location

**Figure-1:**
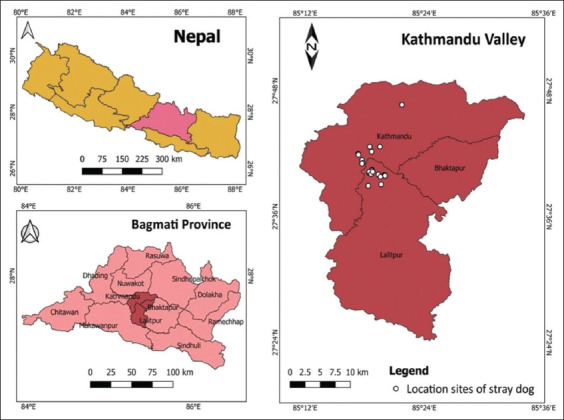
Locality of stray dogs for clinical study and tissue sample.

The study was conducted from 3^rd^ July 2020 to 30^th^ November 2020. The Kathmandu Valley has three districts: Kathmandu, Bhaktapur, and Lalitpur. Kathmandu, Bhaktapur, and Lalitpur cover an area of 395, 119, and 385 sq. km, respectively, in the mid-hill region of the country at an altitude of 1300 m above sea level. It lies between latitude 27°27′N and latitude 27°49′N, and longitude 85°10′E and 85°32′E. According to the dog population survey conducted by Human Society International in March 2016, there were approximately 22,000 street dogs in Kathmandu [17]. Figure-1 shows the sites where stray dogs showing respiratory signs were brought to the animal shelter.

### Clinical examination

Clinical examinations of sick dogs were performed pre-admission and post-admission on a regular schedule according to the shelter protocol. Dogs with signs and symptoms, such as tachypnea, dyspnea, sneezing, coughing, mucopurulent nasal discharge, and exercise intolerance, were considered to have respiratory distress, and clinical observation was continued throughout the treatment. Animals that died during their stay in the shelter were examined grossly and microscopically to detect the underlying disease.

### Postmortem examination and tissue sampling

Necropsy was performed on dead animals that did not respond to treatment. Standard necropsy procedures were followed, and photographs of the target tissues were taken to support the findings of this study. A total of 52 necropsies were performed, and gross pathological findings were described according to tissue alterations observed during the necropsy. The tissues of the lower respiratory system were sampled and preserved in 10% buffer formalin for histopathological evaluation, particularly from grossly altered lungs and associated regional lymph nodes.

## Results

Seventy-six stray dogs were admitted to Animal Nepal for treatment and were presumably diagnosed as respiratory distressed stray dogs on clinical evaluation. The clinical examination included recording vital signs and symptoms. All the admitted dogs underwent the treatment protocol according to the shelter guidelines. Twenty-four dogs recovered and were discharged, whereas 52 dogs died during the treatment.

Necropsy of 52 animals showed variation in gross morphological observation. Lesions in several organs in 27 necropsies suggested non-specific inflammatory causes, whereas gross lung lesions and regional lymph nodes of 25 dogs strongly indicated pulmonary anthracosis (Table-1). Similarly, the histological changes are presented in Table-2.

**Table-1:** Gross changes in the lungs and lymph nodes.

S. No.	Gross pathological observation	Frequency (f)	n (%)
1	Diffused black pigmentation on the external pleural surface	25	100
2	Texture changes in the lung parenchyma	12	48
3	Multiple blackish foci on the dorsal surface of the lungs	8	32
4	Cyst-like raised round areas on the lung	5	20
5	Emphysematous	4	16
6	Consolidation and hemorrhage	13	52
7	Edematous	3	12
8	Lymph node with black particles	25	100

Note: Total number of observations (n)=25

**Table-2:** Microscopic changes in the lungs and lymph nodes.

S. No.	Microscopic features	Frequency (f)	n (%)
1	Presence of exudates with carbon particles in the lung’s parenchyma	25	100
2	Congestion and hemorrhage	15	60
3	Emphysematous changes with broken alveolar septa	16	64
4	Infiltration of mononuclear cells in lymph node	18	72
5	The proliferation of fibrous tissues around bronchioles	7	28
6	Fibrin deposition in alveoli region	9	36
7	Carbon deposition on the medullary region of the lymph node	25	100
8	Deposition of exudates inside the alveolar duct and thickening of the alveolar duct wall	13	52
9	Deposition of exudates in bronchioles	12	48
10	Interstitial pneumonia	17	68

Note: Total number of observations (n)=25

**Figure-2:**
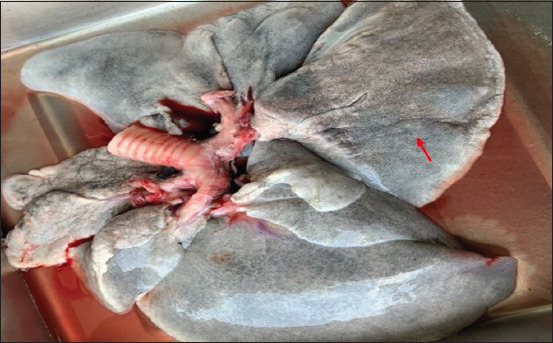
Multiple small or pinpoint black foci scattered throughout the pulmonary parenchyma (red arrow).

**Figure-3:**
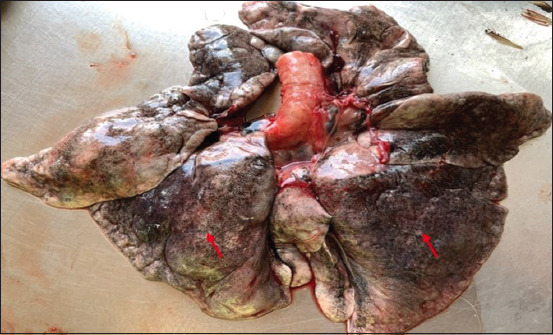
Multiple black foci of variable sizes are present on all the lobes of the lungs (red arrow).

**Figure-4:**
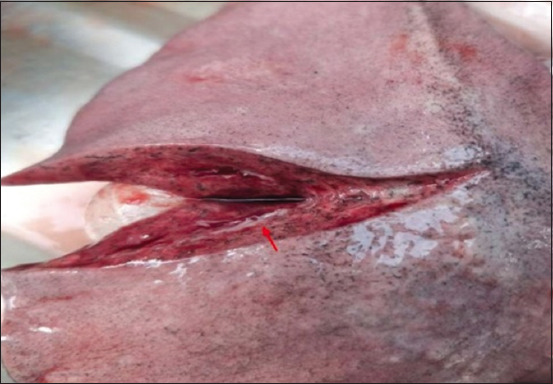
Black-colored foreign particles in the cut surface of the lungs (red arrow).

**Figure-5:**
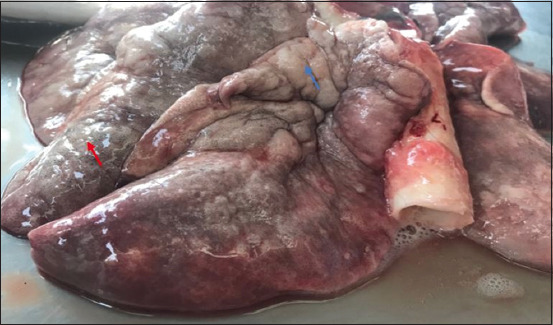
Multiple small/pinpoint foci of blackish discoloration (red arrow) are present on the dorsal surface of the lungs. Some emphysematous areas are also visible (blue arrow).

**Figure-6:**
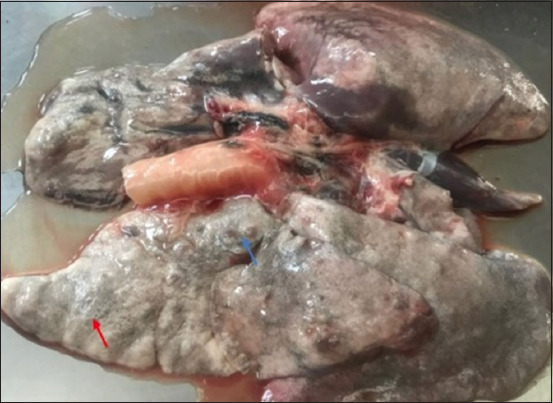
Multiple pinpoint foci of black pigmentation are visible (red arrow). There are few small rounds raised area are also present (blue arrow).

**Figure-7:**
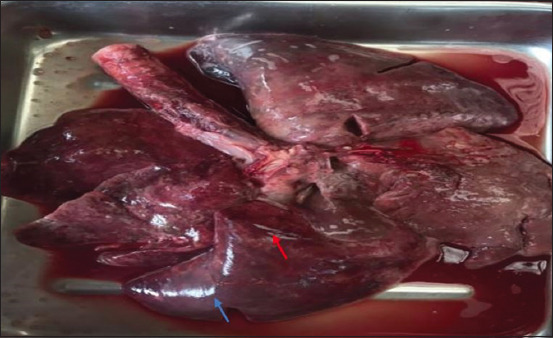
Hemorrhage (red arrow) and congestion (blue arrow) in entire lungs.

**Figure-8:**
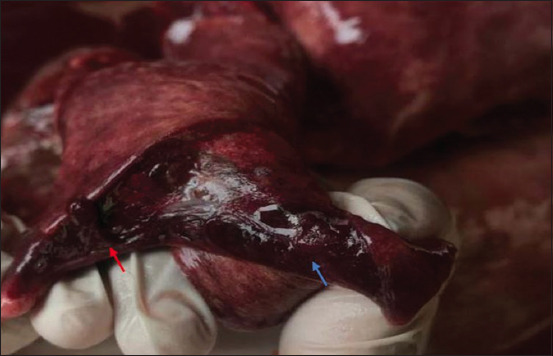
Cut surface of the lung lobe showing hemorrhage (red arrow) and congestion (blue arrow).

**Figure-9:**
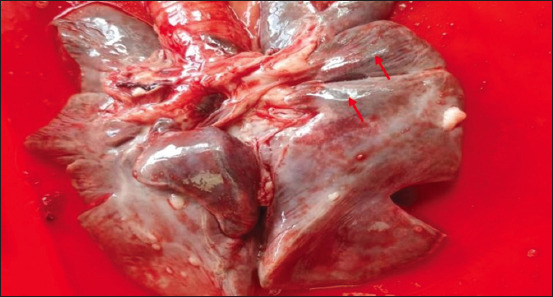
Lungs with black-colored swollen nodules (red arrow).

**Figure-10:**
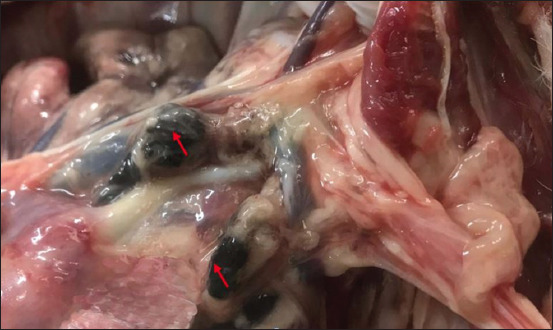
Mediastinal lymph node showing black particles (red arrow).

The morphologic changes recorded most often were lungs sprinkled with black pigments on the external pleural surface (Figure-2), atrophied to hypertrophied, and texture (normal to hard). Multiple black foci of variable sizes and black-colored foreign particles in the cut surface were visible on the lung lobes (Figures-3 and 4). Multiple small/pinpoint foci of blackish discoloration on the dorsal surface of the lungs and emphysematous areas were observed in some of the samples (Figure-5). A few small and raised round areas looked like cysts (Figure 6). In addition, some samples were consolidated and hemorrhaged lungs (Figures-7 and 8) with black swollen nodules (Figure-9) and mediastinal lymph nodes with black pigment deposition (Figure-10) were also observed.

**Figure-11:**
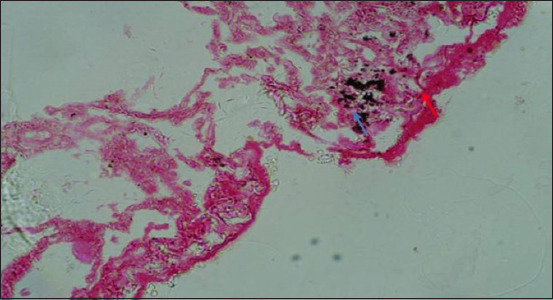
Section showing congestion (red arrow) and carbon deposition (blue arrow) on visceral pleura; H&E stain, 40×.

**Figure-12:**
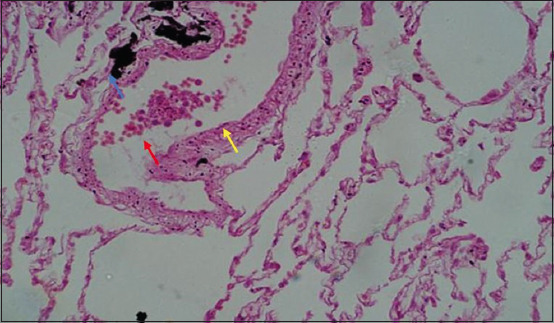
Section of the lung showing the exudates present inside the alveolar duct (red arrow) with carbon particles around the periphery (blue arrow) and thickening of the lining of an alveolar duct (yellow arrow); H&E stain, 40×.

**Figure-13:**
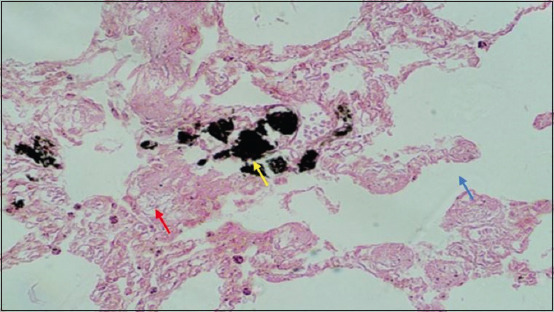
Section of the lung showing emphysematous changes (red arrow) with broken alveolar septa (Blue arrow) along with focal deposition of carbon particles (yellow arrow); H&E stain, 40×.

**Figure-14:**
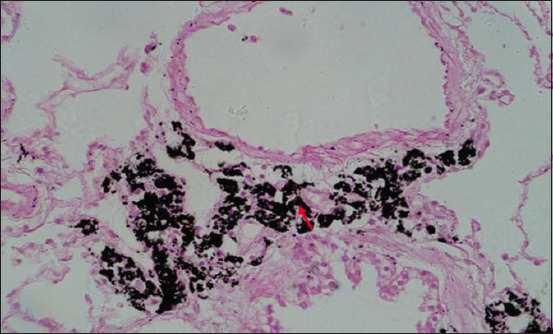
Section showing massive carbon deposition disrupting normal parenchyma of the lung around the periphery of bronchioles (red arrow); H&E stain, 40×.

**Figure-15:**
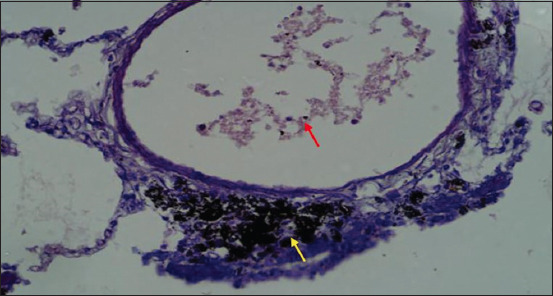
Section of the lung showing deposition of exudates in bronchiole lumen (red arrow) and carbon particles in the periphery of a bronchiole (yellow arrow); H&E stain, 40×.

**Figure-16:**
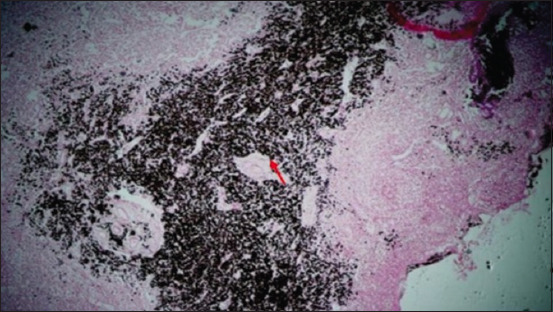
Section showing carbon deposition on the medullary portion of the mediastinal lymph node (peribronchial lymph node) (red arrow); H&E stain, 25×.

**Figure-17:**
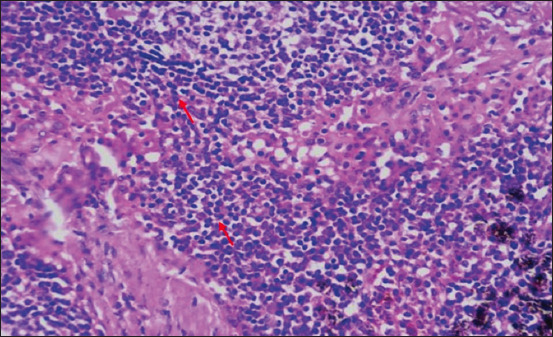
Section showing infiltration of the mononuclear cells on mediastinal lymph node (red arrow); H&E stain, 40×.

**Figure-18:**
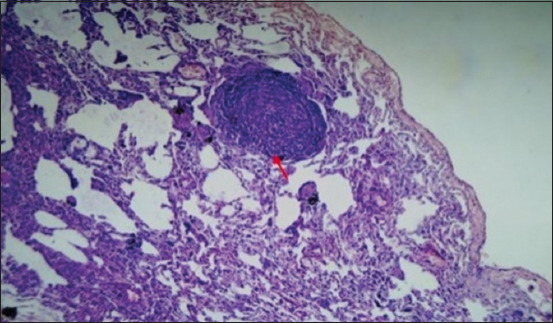
Section showing granulomatous lesion in the alveolar interstitium (red arrow); H&E stain, 25×.

**Figure-19:**
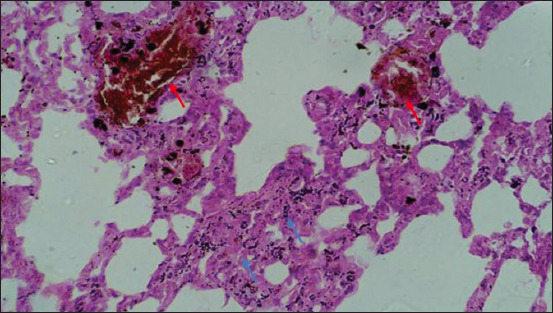
Section of the lung showing areas of congestion (red arrow) with interstitial pneumonia (blue arrow); H&E stain, 40×.

**Figure-20:**
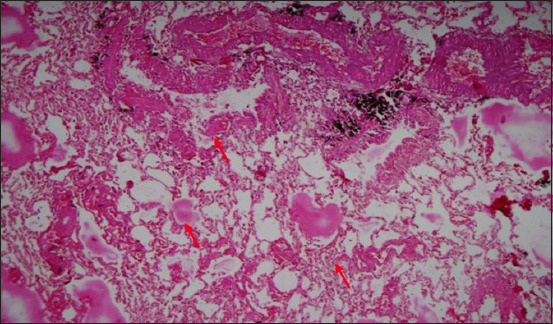
Section of the lung showing the area of fibrin deposit in multiple alveoli (red arrow); H&E stain, 25×.

**Figure-21:**
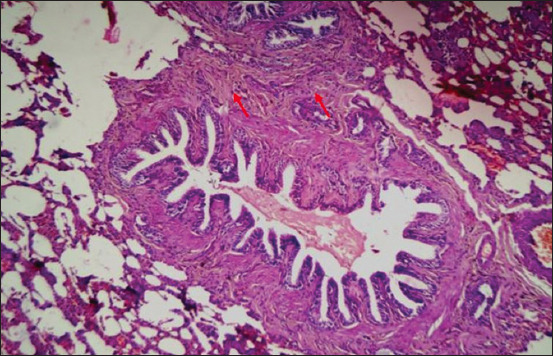
Section showing the area of fibrous tissue proliferation around bronchioles (red arrow); H&E stain; 25×.

**Figure-22:**
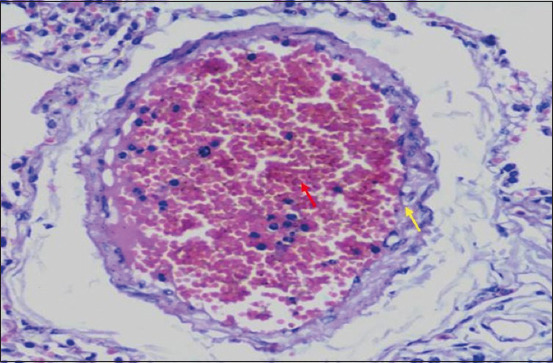
Section showing congested blood vessels (red arrow) and thickening in the wall of a blood vessel (yellow arrow); H&E stain, 40×.

Microscopically, diffused black pigment deposition was observed on most slides. Accumulation of black particles was also observed in the visceral pleural region (Figure-11). Similarly, exudates containing carbon particles were observed inside the alveolar duct around the periphery, and the alveolar duct lining was thickened (Figure-12). Emphysematous changes, along with broken alveolar septa and deposition of black carbon particles in the alveolar septa and around the bronchiole, were also observed in some of the slides (Figure-13). Carbon particles were also observed around the airways, bronchial branches, and pleura (Figure-14). Giant cells and macrophages were observed around the bronchiole, alveoli, and pleural space. Infiltration of macrophages with carbon particles around the periphery was also observed inside the lumen of bronchioles (Figure-15). Carbon particles accumulated even on the bronchioles and lymph nodes. Massive carbon deposition was observed in the medullar region of the lymph node with disruption of normal architecture and infiltration of mononuclear cells (Figures-16 and 17). A granulomatous lesion in the alveolar interstitium was also observed (Figure-18). Interstitial pneumonia with emphysematous changes followed by peripheral pleural congestion was observed (Figure-19). Fibrin deposition with thickening of the bronchiole wall (Figure-20) and proliferation of fibrous tissues around the bronchioles was observed in multiple alveolar regions (Figure-21). Congestive and thickened blood vessel walls were also observed (Figure-22).

## Discussion

This study provides the first documented evidence of pulmonary anthracosis in stray dogs in Nepal, highlighting the threat level from contaminated air in Kathmandu Valley. The previous study has indicated a link between polluted air inhalation and lung cancer and pneumonia development in Nepal [18]. The rapid increase in pulmonary diseases, such as COPD and bronchitis, in rural areas of Nepal is mostly attributable to the use of solid and unclean fuel, smoking, and poor ventilation in the household [1]. In contrast, the rise in pulmonary diseases in urban areas such as Kathmandu is mainly due to outdoor air pollution caused by brick kilns, motor vehicles, and dust storms [1].

Gross morphological examination of the tissues revealed similar lesions resembling pulmonary anthracosis. The lungs exhibited different textures and colors, likely due to the inflammatory response to inhaled dust or carbon particles, which was also observed in a previous study [19]. Consistent black foci and pigmentation in the lungs suggest the inhalation of carbon particles, most likely due to air pollution. In addition, emphysema in the lungs was found, which was likely caused by carbon accumulation in the alveolar spaces. Hemorrhage and congestion were observed in the lungs, possibly due to carbon particles interfering with the hemodynamics of the pulmonary system. The findings of this study support those of the previous study [19–21]. The presence of black pigmentation in the mediastinal lymph nodes was similar to the findings of a previous study, suggesting the accumulation of carbon particles in these nodes as well [14, 21]. Moreover, it appears that the severity of the condition seen in gross observations is influenced by the level of pollution [20].

Similarly, the microscopic study revealed different microscopic changes in the lung tissues and lymph nodes of dogs with respiratory illness, possibly due to exposure to pollutants and carbon particles. Several abnormalities, including deposition of black pigmented particles, congestion, hemorrhage, and exudate accumulation, were observed in various regions of lung tissues and lymph nodes. The suggested changes resulted from an inflammatory process or defense mechanism attempting to remove foreign particles from the respiratory system. This study is supported by previous findings and emphasizes the presence of carbon particles in different lung structures, such as visceral pleura, visceral spaces, alveolar ducts, bronchial areas, and lymph nodes [19, 22–24]. Similar to the previous studies [14, 15, 19, 21], the present study highlights emphysematous alteration, granulomatous lesions, and fibrosis as potential consequences of exposure to carbon particles]. In addition, the microscopic findings observed in the lungs and lymph nodes were consistent with the previous research findings [25, 26].

## Limitations

The gross and microscopic examination results of this study indicate that stray animals living on the streets in Kathmandu are at a significantly high risk of respiratory illness. Our study was conducted on sick dogs presented to the Chobhar animal shelter; therefore, the sample size is limited. Despite the limitations of this study, histological slides will provide insight for future reference in the study of anthracosis. We recommend that risk factors should be incorporated into further research to establish the correlation between PM and the incidence of anthracosis. Similar intervention studies should be conducted to determine the extent to which air pollution can damage human health. Therefore, this study could be helpful for stakeholders involved in public health, pollution, and animal welfare.

## Conclusion

In stray dogs, gross and microscopic alterations in the lungs and lymph nodes provide strong evidence of pulmonary anthracosis. These results highlight a significant threat to both animal and human populations by air quality in Kathmandu. It is, therefore, essential to take immediate measures to address and reduce air pollution to ensure the health and well-being of the Kathmandu people living there.

## Data Availability

The supplementary data can be available from the corresponding author on a reasonable request.

## Authors’ Contributions

PP and ST: Conceived and designed the study, interpreted the gross and histological tissue alterations, and original draft preparation. ST, RBh, BP, SPS, and RK: Collection of samples and necropsy. ST, BP, and RBa: Performed the histology. PP and GR: Developed the research protocol and assisted in interpretation of the data. All authors have read, reviewed, and approved the final manuscript.
